# Supplementary material for: Adapting the coordination of eyes and head to differences in task and environment during fully-mobile visual exploration
Source: PLoS One. 2021 Aug 20;16(8):e0256463. doi: 10.1371/journal.pone.0256463 (PMC8378697; doi:10.1371/journal.pone.0256463)
Supplement: Attachment — Submitted filename: Response to Reviewers.pdf [file pone.0256463.s001.pdf]

# Response to Reviews

Below, we address the reviewer comments point-by-point (our responses follow in italics).

## Reviewer 1's Comments

*We appreciate Reviewer 1's detailed comments, and are glad that they believe the study was "well-motivated and competently conducted". We addressed each of the reviewer's points below.*

Point 1. As the authors described in the text, the two experimental conditions differed not only in the task performed (walk versus search) but also in the environment in which the task was performed (straight versus winding, circuitous path). This should be made explicit in the title of the manuscript, the subtitles of Study 1 and 2 and elsewhere, because the gaze measures might have reflected the difference in the environment, in addition to the task difference.

*We updated the title of the paper to "Adapting the coordination of eyes and head to differences in task and environment during fully-mobile visual exploration", and we updated the subtitles of Study 1 and 2 as well as the results section to note the difference in environment, not just task.*

Point 2. p. 10, line 260: "The walking task took approximately 5 minutes to complete." The authors reported mean speed was 1.16 m/s and the length of the path was 211 m, according to which the walking task should have taken about 3 minutes to complete on average. I wonder why these values are discrepant.
Point 3. Related to Point 2, readers would find it helpful if the authors could provide mean and SD of task duration in walking condition, as well as mean and SD of the distance traveled in search condition.

*We thank the reviewer for pointing out this error. One of the segments (the final east segment) of the walk had the leading digit omitted — it read 35 m instead of 135 m. The total path of 211 m was calculated by adding together the three segments. It now reads correctly as 311 m. Regarding point 3, we added the GPS-derived total path walked by each participant and the total task time to the results section. With the correct values (M distance = 313 m walked / M task time = 268 s = 1.17 m/s) the numbers now correspond (with some minor rounding/averaging differences).*

Point 4. The authors computed the horizontal spread of eye movements and head movements at a single timescale (SD computed over the task duration). I wonder if the information about how the amount of spread increases as a function of the length of time window (i.e., Hurst exponent) may further reveal the difference between the conditions (c.f., Viswanathan et al., 2011, The Physics of Foraging). This is just a comment, not a request.

*We appreciate this suggestion — it's beyond the scope of our questions in this paper, but we will think about incorporating more detail about timescale (and changes over time within the task) in our future work.*

## Reviewer 2's Comments

### Introduction

Please clarify the following sentence: "In other words, observers are more willing to rotate the head when it is going to stay a while." Specifically clarify the "it" that the authors refer to.

*We rephrased the sentence to be more explicit.*

There is a typo on line 89.

*Although it looks like a typo, the author's name is Bernard Marius 't Hart.*

The authors allude to observers "selecting" how much to move the eyes versus the head. Please state whether you believe this to be a conscious cognitive "decision" or if a different conceptual argument is

being made.

*Although it is possible that observers might consciously decide to move their eyes or head a certain way, we believe this reflects a more implicit process. However, we have no data to bear on the degree to which it is conscious or not. We added text to the introduction to make our position clear.*

### Method

I presume that the participants had no motor impairments, but this is not explicitly stated.

*That is correct — we added this to the participants section of both studies.*

Please provide a rationale for why the tracker only focused on the right eye. Is there an argument for a "dominant" eye is participants? If so, was everyone right-handed and right eye dominant?

*Although some (more expensive) mobile eye trackers might track both eyes simultaneously, we did not have access to one. In normal vision (which participants were required to have), both eyes will look in the same spot at the same time under almost all circumstances. If we had a task that required people to look at things incredibly close to the face, we might see differences in eye rotations due to vergence. Even so, these would be complementary between the two eyes and would not impact the metric that we reported (spread). We are not aware of any work suggesting that eye dominance affects the orientation of the eyes to a target, only that participants rely more on the visual input from the dominant eye.*

### Discussion

Please provide a theoretical explanation for your findings (related to the last comment about the introduction).

*We added to the discussion to address the question about conscious control of eye versus head exploration.*

## Reviewer 3's Comments

*We thank Reviewer 3 for commenting that our manuscript was "well-written" and covers an "interesting and important subject". We address the specific comments below.*

Page 2, line 17: ". . . , the eyes within the body,. . . " It would make more sense if it is stated as "the head in relation to the body".

*We reworded the sentence to make it clearer.*

Page 4, line 89-100: I was confused by the semantic use of "spread"; I thought the authors meant to say the range of movement. I can imagine the word "spread" being used by prior related literature and consequently the authors may choose to stick with the convention. Please consider including a brief clarifying statement to alert the readers about the operational use of the word – "spread" – before waiting until the methods section (e.g., page 7, line 162-165).

*We added a definition of "spread" to the introduction before it is first used. Just to clarify, spread is the standard deviation of position, whereas range of movement would be the difference between the minimum and maximum positions (which would reflect the extremes observed within the task, not the typical amount of variability observed in eye/head/gaze position.)*

Page 11, line 288-291: About straightness ratio, I am not certain about the relevance of this parameter being included in the study. Given the walking task and the search task have very different goals and environmental settings, I would be surprised if the there is no difference in straightness ratio between

the two tasks. Perhaps, the authors could include some brief explanation to help the audience better understand the important of this parameter.

*We agree with the reviewer — this is more of a case of trying to numerically describe the expected differences between the tasks rather than to reveal something surprising. We opted to use this metric because it's been used to describe the straightness of reaching as well as walking paths. We kept it in (and explain our rationale in the paper), because it provides a baseline for comparison for future work that might have walking paths that vary along this dimensions (or use tasks that have intermediate levels of straightness compared to the extremes we observed).*

Discussion: The authors, probably influenced by previous literature on visual exploration, seemed to suggest the utilization of head movement even when the task could be achieved by eye movement only may be due to energetic cost. While the authors also presented this unresolved issue as a study limitation. I suggest the authors to consider the other sensory systems (e.g., vestibular, neck proprioception) that may be involved during head movement as to potentially explain why the involvement of head movement may be beneficial while performing these tasks.

*We appreciate the suggestion, and acknowledge this possibility in the discussion.*

Figure 1 and Figure 2: These two figures are incorrectly placed and referred.

*Thank you – we corrected the figure ordering.*

References: Please check your citation format. Some are incorrect.

*We fixed the citation format.*
